# Ultrasound-Guided Percutaneous Radiofrequency Thermal Neuroablation for the Treatment of Adductor and Rectus Femoris Spasticity

**DOI:** 10.7759/cureus.33422

**Published:** 2023-01-05

**Authors:** Adriana Pascoal, Carolina Lourenço, Filipe N Ermida, Ana Costa, José Luís Carvalho

**Affiliations:** 1 Physical Medicine and Rehabilitation, Centro de Medicina de Reabilitação da Região Centro - Rovisco Pais, Cantanhede, PRT; 2 Physical Medicine and Rehabilitation, Centro de Medicina de Reabilitação da Região Centro - Rovisco Pais, Tocha, PRT; 3 Centro de Reabilitação do Norte, Centro Hospitalar Vila Nova de Gaia/Espinho, Vila Nova de Gaia, PRT

**Keywords:** paraplegia, spinal cord injury, spasticity, ultrasound guided, radiofrequency thermic neuroablation

## Abstract

Spasticity is a very frequent complication of spinal cord injury (SCI) that can limit activities of daily living, reduce the quality of life, and augment caregiver burden. This problem has many treatment options that should be selected according to the clinical and functional scenario.

This case study presents a 60-year-old female patient with complete spastic paraplegia after a spinal stroke. Spasticity interfered with activities of daily living, mainly with intermittent catheterization and transfers, and botulinum toxin injections failed to efficiently treat this issue. It was decided to perform an ultrasound-guided radiofrequency thermal ablation of the anterior and posterior branches of the obturator nerve and motor branches to the rectus femoris of the femoral nerve to treat the adductors and rectus femoris spasticity. One year after the radiofrequency treatment, the patient showed considerably reduced spasticity, allowing her caregiver to do transfers and easier intermittent urinary catheterizations.

Nerve radiofrequency thermal ablation has the potential to be an effective therapy in lower limb spasticity, with long-lasting effects.

## Introduction

Spasticity is a very common complication after spinal cord injury (SCI), characterized by a velocity-dependent increase in muscle resistance to passive stretching, leading to stiffness and abnormal limb posturing [1].

A comprehensive clinical evaluation should include other differential diagnoses for increased muscle tone, identification of potential triggers (such as pain or infection), clinical measures such as the Modified Ashworth Scale (MAS), and the impact on functionality. When minimally invasive procedures are considered, clear objectives for the patient should be defined and, if applicable, for the respective caregiver [2].

Physical consequences after spasticity, such as abnormal limb posture, can functionally limit activities of daily living, reduce the quality of life, and increase caregiver burden. This problem has many treatment options that should be selected according to the clinical and functional scenario. Treatment can include a range of non-pharmacological options (extracorporeal shock wave therapy, stretching techniques, neuromuscular and functional electrical stimulation, transcutaneous electrical nerve stimulation, transcranial direct current stimulation, etc.), pharmacologic options (baclofen, tizanidine, dantrolene, benzodiazepines, gabapentin, botulinum toxin, etc.), and various surgical techniques (lengthening techniques, tendon transfers, tenotomy, neurectomies, rhizotomy, peripheral neurotomy, etc.) [3]. Other options include selective neurolysis, including alcohol and phenol, or botulinum toxin chemodenervation [4]. To our knowledge, there is only one study with peripheral neuro ablation using thermal percutaneous radiofrequency [5] and scant studies using cryoablation [4].

Neuroablation using thermal percutaneous radiofrequency consists of the passage of radiofrequency currents across an electrode close to a nociceptive nerve to stop the pain signals. The continuous radiofrequency currents produce thermal energy that creates a circumscribed area of tissue damage. It can be applied to several kinds of tissues, including peripheral nerves, allowing the treatment of different pain syndromes. The absolute contraindications of this procedure include patient rejection, increased intracranial pressure, and local infection. The relative contraindications include bacteremia and atypical congenital or surgical anatomy. The most relevant reported complication is neuropathic pain (when the peripheral nerve has a sensory component). Adverse effects and complications are very rare and can be diminished by several factors such as proper technique, image guidance, asepsis, and procedural skill [6].

In this article, the authors describe a case of adductor and rectus femoris spasticity treated with neuro ablation by thermal percutaneous radiofrequency under ultrasound guidance.

## Case presentation

A 60-year-old female patient was a victim of a spinal stroke that led to paraplegia with an American Spinal Injury Association (ASIA) Impairment Scale (AIS) grade A, T2 neurological level of injury. The International Standards for Neurological Classification of Spinal Cord Injury (ISNCSCI), commonly referred to as the ASIA Exam, was developed by the American Spinal Injury Association (ASIA) as a universal classification tool for spinal cord injuries based on a standardized sensory and motor assessment [7].

The patient had spasticity in the lower limbs, making intermittent catheterization and transfers difficult. Therefore, these activities of daily living were performed by her caregiver with great difficulty.

Seven months after the spinal stroke, she was observed in a physiatry outpatient appointment. The physical exam revealed spasticity in the adductor group and rectus femoris grade 3 bilaterally, in the MAS. The adductor spasticity interfered with bladder management and the rectus femoris spasticity restricted knee flexion during transfers. Because of the functional limitation associated with spasticity, she underwent treatment with a botulinum toxin A with the following dose distribution: 200 units in each adductor longus, 300 in each adductor magnus, and 150 in each rectus femoris. After the botulinum toxin injections, the patient integrated a rehabilitation program with physical therapy focusing on stretching both the hip adductors and knee extensors. The treatment improved spasticity and facilitated care, however, the effect was satisfactory for only three weeks.

So, after excluding other causes of spasticity with insufficient response to therapy, such as infection, pain, or constipation, a different treatment was decided.

Pumps can be surgically implanted to deliver intrathecal baclofen for treating the spasticity after spinal cord injury and there is level 1b evidence, from two randomized controlled trials, that intrathecal baclofen is a cost-effective intervention [8]. However, after explaining this therapeutic option, the patient rejected the baclofen pump implantation.

After the explanation of the different treatment hypotheses and obtaining the patient’s consent, it was decided to perform a radiofrequency (RF) thermal neurolysis four months after the botulinum toxin treatment.

Procedure

Before the procedure, the patient was positioned supine, and the anterior and posterior branches of the obturator and motor branches to the rectus femoris of the femoral nerves were identified using a 3.7-13 MHz linear probe (Logiq™ P8 ultrasound machine, GE Healthcare, Chicago, Illinois, United States). The anterior branch of the obturator nerve was identified in the fascial plane between the adductor longus and adductor brevis, the posterior branch of the obturator nerves between the adductor brevis and adductor magnus and the femoral nerve at the femoral neurovascular bundle, over iliacus muscle and proximal to the origin of the direct head of the rectus femoris.

**Figure 1:**
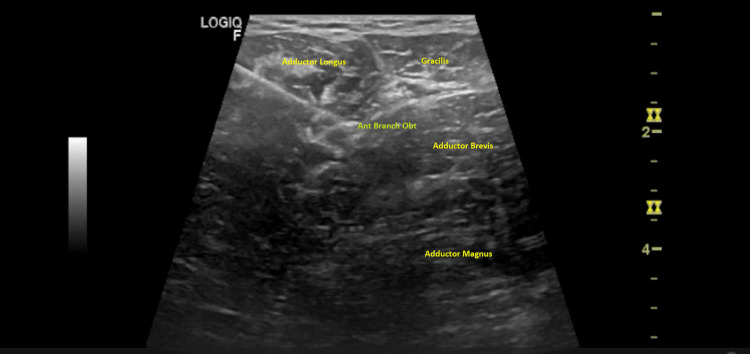
Anterior branch of obturator nerve Anterior branch of the obturator nerve in the fascial plane between the adductor longus and adductor brevis. Tip of the needle near the anterior branch of the obturator nerve (Ant Branch Obt).

After aseptic measures, a specific 22-gauge radiofrequency cannula - 10cm x 10mm x 22ga (0,7mm) - was used, placing the tip close to the nerve (Figure 1).

Considering that the patient had a complete spinal cord injury, with anesthesia at the lower limb level, it was decided not to perform a prior anesthetic block. However, before the ablation technique, a motor test was performed to confirm the electrode proper placement. Once the radiofrequency cannula tip was positioned under ultrasound guidance close to the target (anterior and posterior branches of the obturator and motor branches to the rectus femoris of the femoral nerves), the stylet was replaced with a radiofrequency electrode, connecting it to the radiofrequency generator. The final tip position was confirmed when motor stimulation induced muscle contraction of the rectus femoris and hip adductors. 

Two cycles for each nerve of percutaneous radiofrequency thermal ablation were conducted with the following parameters: 100 V, 80ºC, for 2 minutes. There was an immediate response in muscle tone reduction of rectus femoris and adductors after the procedure, from grade 3 to grade 1 in the MAS. After the procedure, the patient maintained a rehabilitation plan that included spasticity control (focusing on hip adductors and knee extensors stretching) and activities of daily living training.

Results

The procedure had no complications. A follow-up was scheduled at three and twelve months to evaluate its medium- and long-term benefits. Three and twelve months after the procedure, the patient-maintained bilateral spasticity in the adductor group and rectus femoris up to grade 1 on the MAS, and the patient and her caregiver were satisfied with the outcome.

The patient continued to need the support of a third person to perform the transfers and intermittent catheterization. After the procedure, the caregiver reported easier transfers and vesical intermittent catheterization without difficulty.

## Discussion

An integrated goal-centered approach is essential for successful care for spasticity. Peripheral nerve ablation cannot be a first-line solution in many situations, such as when the return of functional movement is possible, the spasticity is useful for activities such as transfers or gait and other more conservative therapeutic measures can treat the spasticity. 

In our clinical case, the botulinum toxin treatment facilitated care for only three weeks. The effects of botulinum toxin are not permanent and the patient experience varies. Clinical studies have shown a significant reduction in spasticity with peak effects at four to six weeks and effectiveness decline gradually thereafter. An electronic survey to provide an evaluation of how patients living with spasticity experience the therapeutic effects of botulinum toxin treatment showed that 6% of the patients reported symptom re-emergence within two months [9]. However, there is a lack of literature studying the impact of botulinum toxin on function and quality of life in persons with spinal cord injuries [10]. 

A spinal cord injury patient with severe spasticity, non-responsive to antispastic oral drugs, botulinum toxin, and other conservative therapeutic options can benefit from an intrathecal baclofen pump. Baclofen (oral or intrathecal) is a proven useful pharmacologic agent for patients with spasticity. However, it has a short therapeutic window that requires special dose monitoring. Its toxicity and withdrawal are life-threatening complications and the pump and/or the intrathecal catheter may also be the source of complications such as intrathecal migration and infection [11].

To our knowledge, the only study reporting treatment of lower limb spasticity with neuroablation using percutaneous thermal radiofrequency was published in 1987 and did not use image guidance. The authors used a temperature-monitoring needle electrode system to localize the nerves by stimulation. The obturator neurotomy was performed in the most proximal portion accessible, receiving four cycles lasting 60 seconds between 65 and 70 degrees Celsius [5]. This procedure has quite a few advantages, such as the simplicity of the method and the absence of postprocedural limitations in rehabilitation care. When the procedure is performed with ultrasound guidance, it is potentially safer, considering that the medical professional can see the adjacent noble structures and control peripheral tissue damage (a great advantage facing chemical neurolysis, which cannot control the area of destruction because of the unpredictable spread pattern).

Cryoneurolysis is an alternative technique of neuroablation. This modality is not associated with neuroma formation or hyperalgesia, an advantage versus surgical sectioning, radiofrequency thermal ablation, or chemical neurolysis. However, cryoneurolysis has the disadvantage of reduced effect duration [6]. Spinal cord injury patients with motor sparing have an increased likelihood of below-level neuropathic pain, suggesting a protection factor for those with complete injuries, such as our patient [12]. Though this type of technology was not available in our hospital, it can be considered an option for spasticity treatment when neuropathic pain is a crucial issue. 

## Conclusions

Nerve radiofrequency thermal ablation has the potential to be an effective therapy in lower limb spasticity, with long-lasting effects. It is also a simple and minimally invasive procedure. Despite the advantages of this treatment, it cannot be a solution for all patients with spasticity. An integrated goal-centered approach is essential for successful care for spinal cord injury patients. Therefore, performing a functional prognosis as correctly as possible, before a potentially definitive therapeutic decision, is crucial.

A standardized approach should be used in research to evaluate the effects of this treatment in spasticity and compare it with more traditional options, such as botulinum toxin and oral and intrathecal baclofen.
